# Technology-induced job loss risk, disability and all-cause mortality in Norway

**DOI:** 10.1136/oemed-2021-107598

**Published:** 2021-09-24

**Authors:** Bernt Bratsberg, Ole Rogeberg, Vegard Skirbekk

**Affiliations:** 1 Frisch Centre for Economic Research, Oslo, Norway; 2 Centre for Fertility and Health, Norwegian Institute of Public Health, Oslo, Norway; 3 Columbia Aging Center, Columbia University Medical Center, New York, New York, USA; 4 Norwegian National Advisory Unit on Ageing and Health, Vestfold Hospital Trust, Tønsberg, Norway; 5 Department of Psychology, University of Oslo, Oslo, Norway

**Keywords:** disability, mortality, longitudinal studies

## Abstract

**Background:**

Ongoing shifts in economic structure from automation and globalisation can affect employment and mortality, yet these relations are not well described.

**Objective:**

We assess whether long-term employment and health outcomes relate systematically to structural change in the labour market, using the occupational Routine Task Intensity (RTI) score as indicator of exposure is to risks of outsourcing and technology-induced job loss.

**Methods:**

Using a cohort design and administrative data with national population coverage, we categorise all Norwegian employees in 2003 by the RTI score of their occupation and examine how this score correlates with employment and health outcomes measured in 2018 and 2019. The study sample counts 416 003 men and 376 413 women aged 33–52 in 2003.

**Results:**

The occupational RTI score at baseline is robustly associated with long-term employment, disability and mortality outcomes. Raw correlations are reduced after adjustment for potential confounders, but associations remain substantial in models controlling for individual covariates and in sibling comparisons. Working in an occupation with RTI score 1 SD above the mean in 2003 is associated with a raised probability of being deceased in 2019 of 0.24 percentage points (95% CI: 0.18 to 0.30) for men and 0.13 percentage points (95% CI: 0.02 to 0.24) for women, corresponding to raised mortality rates of 6.7% and 5.5%.

**Conclusions:**

Individuals in occupations characterised by high routine intensity are less likely to remain employed in the long term, and have higher rates of disability and mortality.

**Key‌ ‌messages**

**What is already known about this subject?**

- Job loss and precarious employment is known to be related to negative health outcomes. Yet, there have been insufficient large-scale population level longitudinal analyses focusing on how exposed the job is to technology-induced displacement and its health and social effects.

**What are the new findings?**

- We find strong correlations between job loss risk at the occupational level in 2003 and employment, disability and mortality outcomes measured 16 years later.

**How might this impact on policy or clinical practice in the foreseeable future?**

- Retraining programs and career advice may help improve employment adaptability of those holding jobs at risk of displacement.

Stable and secure employment tends to be associated with better health and longer lives.^1 2^ Holding work that is perceived to be precarious or undesired could affect lifestyles and health risk behaviours.^3 4^ While associations between type of work and job loss can reflect selection into jobs, research designs that largely avoid individual-level confounding have been found to reveal health effects of job loss—as in a recent paper comparing cause-specific mortality following unemployment from stable, downsized and closed workplaces.^5^ As a result, public health outcomes and health inequalities may be adversely affected by ongoing and interlaced processes of globalisation and automation currently altering the structure of Western labour markets, processes that are hollowing out employment shares of mid-income occupations and causing job loss in trade-exposed regions.^6^ Regional malaise relating to such economic disruptions has been argued to raise mortality from suicide, drug and alcohol use,^7^ though this claim remains contested.^8^ While the projected impact of technological changes on labour markets varies across studies,^9 10^ many expect these economic changes to continue or even accelerate—and encompass larger shares of the economy. One assessment by the World Bank^11^ suggests that one half of all jobs held today are at risk of disappearing due to automation. Technology-induced job losses could further disproportionally affect certain demographic groups. In highly gender-stratified economies, which includes Norway, the implications for industrial employment could disproportionally affect men.^12^

There are several reasons why technology-induced job loss can relate to health outcomes.^13^ Holding an occupation that is being phased out over time increases the risk of employment loss and makes re-employment harder since job openings within the same occupation will tend to become scarce. Unemployment has been found to be associated with worsening mental health,^14^ while job insecurity may affect those who remain employed. Having a job where one has a higher risk of being laid off can cause stress and greater risk of anxiety and depression.^15^ Perceived job security and stressful working conditions are associated with the risk of new technologies displacing one’s job.^16–18^ Employees whose jobs face automation may be more likely to fear job displacement,^19^ and studies report associations between low job security and worsened health conditions for employees and their families,^20 21^ as well as fear of job displacement and reduced mental health.^22 23^ Norwegian data indicate a rise in the use of antidepressants and anxiety-reducing prescription drugs several months before a job loss occurs.^24^ Job loss is strongly associated with mental health and health risk behaviours, such as cigarette smoking, alcohol intake and physical inactivity.^25–27^

While this suggests that a link between economic structural change and health is plausible, the claimed link between unemployment/labour market outcomes and health is still contested. While some read the evidence as strongly supporting harmful causal effects of job loss on physical health and mortality,^28^ others suggest this mainly reflects reverse causality whereby poor health increases the probability of unemployment.^29^

The objective of the current study is to examine how structural economic risk at the occupational level relates to long-term health outcomes of employees, using large-scale administrative data registers from Norway with full population coverage for the 2003–2019 period and detailed information on occupation, individual background characteristics and long-term health-related outcomes.

## Methods

### Data

Our main analysis uses a cohort design to examine whether employment, disability and mortality in 2018 and 2019 is systematically related to the structural risk facing an individual’s occupation in 2003. The structural risk is measured using a widely used occupation-level indicator of automation and outsourcing risk. Individual-level covariates and a fixed effect model comparing same-sex siblings are used to adjust for confounders.

The study covers all individuals with registered employment in November 2003 and observes employment and disability status in 2018 and mortality status in 2019. Administrative registers with full population coverage were linked to combine employment records with pre-tax earnings and occupational code, educational attainment (normed years), demographic information (gender, year of birth, identifiers for civil status and children), social transfers (disability pension) and mortality. The analysis data cover the period 2003–2019 (with disability pensions and employment observed through 2018), as 2003 was the first year with occupation codes (the ISCO-88 standard) in the Norwegian employer–employee register. Our data extract covers workers in 335 occupations at the four-digit level.

As an indicator of automation and outsourcing risk at the occupational level, we use the Routine Task Intensity (RTI) index,^30–32^ linked to occupations using occupational code cross-walks from the standards used by the O*NET Database which includes characteristics of job tasks across occupations. The RTI index is a weighted sum of selected job characteristics measured in the O*NET data, intended to capture the extent to which an occupation is characterised by routine cognitive or physical tasks that can potentially be automated or outsourced (see Mihaylov and Tijdens^33^ for details on the RTI construction used). The index is computed as the sum of the occupation’s scores on the routine manual and routine cognitive task scales, and subtracts the scores on three non-routine task scales (manual, analytical and interpersonal). To facilitate interpretation of results, we standardise the index to have mean 0 and SD 1 in the 2003 workforce.

Consistent with the underlying theory of skill biased technological change, past research has found declining employment in occupations with higher RTI scores–leading to a hollowing-out of employment in medium-pay occupations in both the USA^6^ and Europe.^30^ To match the year of the worker extract, we use the 5.0 (2003) release of the O*NET Database.

The analyses are designed to examine how the structural risk score of employee occupation at baseline in 2003 predicts the probability of (a) remaining in employment in 2018 and (b) developing serious health issues—as measured by medical disability in 2018 and age-adjusted mortality in 2019. Occupation-level indicators of structural risk are used in place of observed individual job displacement so as to reduce bias from spurious associations reflecting selection into unemployment.^5^ To further adjust for selection into occupations, we also analyse the data using additional individual-level variables and a same-sex family fixed effect design to control for unmeasured, time-invariant family-level confounders.

We analyse three separate outcomes. Employment is defined as having registered employment with annual earnings above 1 G, the base unit of the Norwegian pension system and the minimum earnings for pension rights. As indicators of long-term health and mortality at the individual level, we use data indicating whether an individual received a permanent disability pension or was registered as deceased in the population registry. The criteria for receiving disability pension are that an individual has suffered a permanent reduction in earnings ability due to serious illness or injury. Diagnoses vary by age and sex, but mental health and musculoskeletal problems jointly account for a majority of cases in most age groups for both sexes, as is the case in the other Nordic countries.^34 35^

We study the long-term health outcomes correlated with employment in occupations with varying exposure to structural change in labour markets. Our main analysis sample is therefore restricted to wage earners in their prime earnings age with a valid record (ie, non-zero hours and wages) in November 2003 in the employer–employee register and we exclude those who were disability pension recipients in 2003. Because we can follow the earnings and social security history of these workers for 15 years (ie, through 2018), we limit the data extract to those aged 33–52 in 2003. People aged 53 or more at baseline would not have disability status observed in 2018, as these are converted to old-age pensions at age 67. To ensure that we have links to parents and complete records of mortality, we further restrict the extract to Norwegian-born individuals with two Norwegian-born parents. Prior research shows that, among immigrants to Norway 1967–2002, 60% had outmigrated within 10 years of arrival.^36^ The data extract we use consists of 416 003 men and 376 413 women born between 1951 and 1970. From these records, we construct subsamples for the purpose of conducting sibling comparisons, identifying 186 369 men with a brother and 158 524 women with a sister in the employee extract.

Further details on variable definitions and estimation code are available in the online supplemental file 1.

10.1136/oemed-2021-107598.supp1 Supplementary data

### Statistical analyses and outcomes

We assess associations between occupational risk scores and long-term outcomes at both the occupational and individual levels. For the occupation-level analysis, each observation consists of an occupation, its 2003 standardised RTI score, the number of employees working in the occupation in 2003 and the average employment, disability and mortality of these employees measured in 2018–2019. In the occupation-level analysis, the associations between the RTI score of the occupation and the three outcomes are assessed using separate linear regressions with observation weights equal to the number of employees in each occupation, superimposed on a bubble chart—a scatter plot where each occupation is represented as a circle scaled to show the relative occupational size in 2003.

In the analyses using individual-level data, we assess the same associations using linear probability models and three separate model specifications that allow for varying degree of confounder adjustment. The first model uses no controls, closely matching the occupation-level analysis. The second model adds controls for age (indicator variables for age), educational attainment (indicators for 13 levels), civil status (single as opposed to married/cohabitant) and childlessness. Age and educational attainment dummies are used to avoid restrictive functional forms. The third model adds family fixed effects, which means that each family has its own unique intercept. This identifies the coefficients by assessing whether sibling differences in occupational RTI scores predict sibling differences in long-term outcomes. By using within-family variation only, this effectively controls for all (observed and unobserved) time-invariant confounders at the family level (eg, family and socioeconomic background, shared genetic and environmental influences).

All models are estimated separately for each combination of sex and outcome, with SEs in the individual-level analyses clustered within occupations.

We also re-estimate the confounder control and within-family models with an interaction term allowing the RTI coefficient to differ linearly by years of education, so as to assess whether associations differ by socioeconomic status of employees.

Finally, as a robustness exercise, we perform the same set of analyses using the Frey-Osborne (FO) index in place of the RTI index as the measure of structural risk. While the two measures would be expected to correlate, the FO index was developed in 2012 and aimed to more narrowly reflect the probability that expected advances in machine-learning techniques would make it possible to automate the tasks involved in different occupations over the coming decades.^31^

## Results

The main analysis sample consists of 416 003 men and 376 413 women (table 1). The average age and educational attainment of the men and women are similar, but men are 1% point more likely to be single and 7% points more likely to be childless at baseline. For the family fixed effect models, only individuals with a same-sex sibling in the data will contribute to the estimation, reducing the sample sizes to 186 369 men (‘Brothers’) and 158 524 women (‘Sisters’). Relative to the full sample, the individuals in the sibling samples are about 1% point less likely to live alone and are more likely to have children, with slightly lower long-term disability retirement and mortality at follow-up.

**Table 1:** Descriptive statistics, analyses samples

	Men		Women	
	All	Brothers	All	Sisters
	(1)	(2)	(3)	(4)
Observations	416 003	186 369	376 413	158 524
Number of families		83 715		71 647
Age	42.1	42.3	42.3	42.3
Education (years)	13.4	13.4	13.5	13.4
Single (%)	29.2	28.1	28.3	27.5
Childless (%)	18.6	14.9	11.8	10.8
Employment per 100, 2018	86.0	87.8	81.9	84.0
Disability pensions per 100, 2018	10.5	9.9	17.8	17.0
Mortality per 1000, 2019	35.6	32.5	23.7	22.7

Samples are drawn from the November 2003 employment file of the Norwegian welfare administration and are limited to wage earners age 33–52. Unless otherwise stated, descriptive statistics are measured in 2003. Employment and disability statistics are conditional on survival until 2019. See text for further details on sample restrictions.

**Figure 1:**
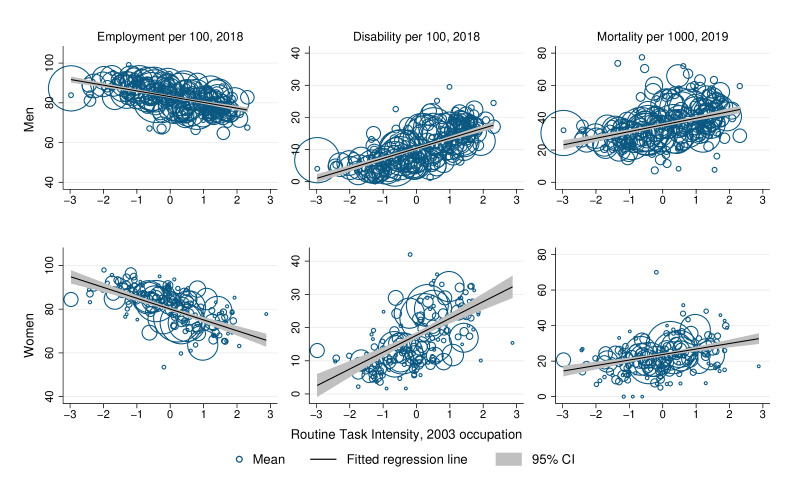
Occupational risk score 2003 and employment, disability and mortality 2018/2019. Scatter points show the average outcome in 2018 or 2019 versus the Routine Task Intensity index for each of 246 (men) and 185 (women) 2003 occupations. Occupations with higher RTI scores are expected to be more influenced by automation and globalisation. Scatter points are weighted by the observation count of the 2003 occupation; cells with fewer than 100 observations are omitted from the figure. The Routine Task Intensity index is standardised to have mean 0 and SD 1 in the 2003 workforce. Slope (95% CI) of regression lines are −3.00 (−3.44 to –2.55), 3.22 (2.84 to 3.62) and 4.38 (3.52 to 5.24) in the top panels, and −5.07 (−6.00 to –4.14), 5.18 (4.12 to 6.25) and 3.20 (2.25 to 4.14) in the bottom panels. RTI, Routine Task Intensity.

The occupation-level analysis presents unadjusted estimates, and shows higher RTI scores at baseline robustly associated with poorer long-term average outcomes for employees of both sexes (figure 1). The results are shown separately for each combination of sex and outcome variable, with a regression line and 95% CI superimposed on a bubble plot of standardised RTI scores against average long-term outcomes of each occupation’s employees. The size of each bubble reflects the occupation’s employment share at baseline, and thus also the observation’s weight in the regression.

Individual-level analyses allow for confounder-adjusted estimates, with results shown in table 2. Point estimates are substantial for all model–sex–outcome combinations, but the magnitude of the coefficients is substantially reduced when controlling for individual-level observables. Note that the point estimates remain relatively unchanged as family fixed effects are added, although precision is reduced as the estimates use a smaller sample and only exploit the within-family variation in occupational risk score. The small change in estimates is consistent with our observed covariates capturing much of the compositional differences in employee characteristics across occupations.

**Table 2:** Regression results, coefficient of Routine Task Intensity index of 2003 occupation

	Men			Women		
	Model w/o controls	Model with controls	Sibling model	Model w/o controls	Model with controls	Sibling model
	(1)	(2)	(3)	(4)	(5)	(6)
Dependent variable						
Employment per 100, 2018	−2.8	−2.0	−1.5	−5.0	−2.1	−1.6
	(−3.5 to –2.1)	(−2.3 to –1.6)	(−1.8 to –1.2)	(−6.9 to –3.1)	(−3.2 to –1.0)	(−2.3 to –0.9)
Disability per 100, 2018	3.3	1.9	1.6	5.3	2.0	1.7
	(2.5 to 4.1)	(1.5 to 2.4)	(1.2 to 2.0)	(3.2 to 7.4)	(0.6 to 3.4)	(0.7 to 2.6)
Mortality per 1000, 2019	4.4	2.4	2.4	3.3	1.3	1.4
	(3.2 to 5.6)	(1.8 to 3.0)	(1.2 to 3.6)	(1.7 to 4.9)	(0.2 to 2.4)	(−0.1 to 2.9)
Observations	416 003	416 003	186 369	376 413	376 413	158 524
Number of families			83 715			71 647
Control variables	None	Age, education, civil status, childless	Age, education, civil status, childless, family fixed effects	None	Age, education, civil status, childless	Age, education, civil status, childless, family fixed effects
Sample	All	All	Brothers	All	All	Sisters

Table entries give change in dependent variable from a 1 SD increase in the Routine Task Intensity (RTI) index of 2003 occupation. Higher RTI scores reflect a greater susceptibility to the effects of automation and globalisation. 95% CIs are reported in brackets; SEs are clustered within occupations. Models in columns (2), (3), (5) and (6) include indicator variables for 20 ages and 13 levels of educational attainment, as well as indicator variables for single status and childlessness in 2003. Employment and disability outcomes are conditional on survival until 2019.

The coefficient of the standardised RTI score expresses the outcome difference associated with a 1 SD change in occupational risk score. To illustrate, in the most cautious model for men (table 2, sibling model), an individual whose initial occupation had an RTI score 1 SD above his brother would be predicted to have a 1.5 percentage points (95% CI: 1.2 to 1.8) point reduced probability of being observed in employment, a 1.6 percentage points (95% CI: 1.2 to 2.0) raised probability of being observed with disability pension and a 0.2 percentage point (95% CI: 0.1 to 0.4) point higher probability of being dead. Our most conservative estimate of the effect on mortality comes from the confounder-control model estimated using the full sample. Evaluated at the sample mean, the point estimate from this model implies that a 1 SD RTI score difference is associated with a 6.7% higher mortality rate among men and 5.5% higher mortality rate among women.

**Figure 2:**
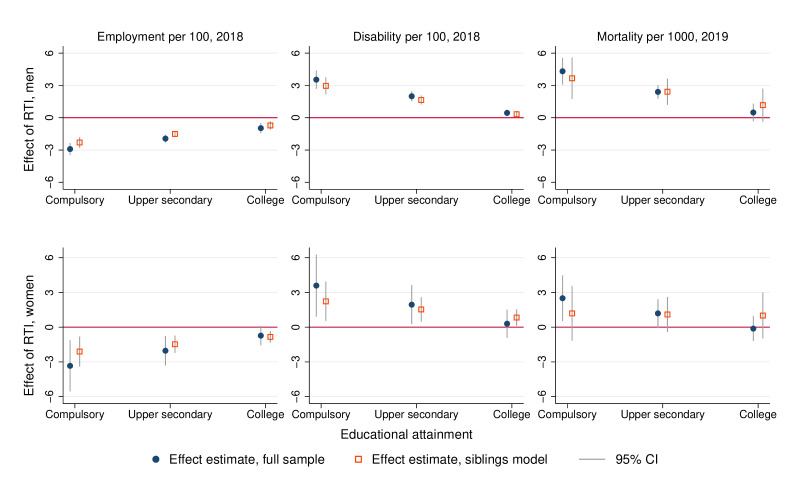
Effect estimates of RTI on employment and health outcomes by educational attainment. Scatter points show the estimated effects of a 1 SD increase in the Routine Task Intensity index of 2003 occupation, evaluated at educational attainments of compulsory schooling, completed upper secondary and college. Estimates are based on regression models where attainment is interacted with RTI. Regression models control for age (20 levels), single status and childlessness in 2003. Sibling models add family fixed effects. SEs are clustered within 2003 occupation. RTI, Routine Task Intensity.

Allowing the RTI coefficient to vary by educational attainment finds a consistent pattern across sex–outcome combinations of coefficients taking a larger absolute value for those with less education, though several of the coefficients are imprecisely estimated (table 3). To simplify interpretation and comparison of these results, we evaluate the RTI coefficient at three different levels of attainment; compulsory schooling, completed upper secondary education and college degree (figure 2).

**Table 3:** Coefficient of interaction term between Routine Task Intensity index of 2003 occupation and educational attainment

Dependent variable	Employment per 100, 2018		Disability per 100, 2018		Mortality per 1000, 2019	
Sample	All	Siblings	All	Siblings	All	Siblings
	(1)	(2)	(3)	(4)	(5)	(6)
Men	0.3	0.3	−0.5	−0.4	−0.6	−0.4
	(0.2 to 0.5)	(0.2 to 0.4)	(−0.6 to –0.3)	(−0.5 to –0.3)	(−0.9 to –0.3)	(−0.9 to 0.0)
Women	0.4	0.2	−0.5	−0.3	−0.4	−0.1
	(0.1 to 0.8)	(−0.0 to 0.4)	(−0.9 to –0.1)	(−0.5 to 0.0)	(−0.7 to –0.1)	(−0.6 to 0.4)
Control variables	RTI, education, age, civil status, childless	RTI, education, age, civil status, childless, family fixed effects	RTI, education, age, civil status, childless	RTI, education, age, civil status, childless, family fixed effects	RTI, education, age, civil status, childless	RTI, education, age, civil status, childless, family fixed effects

Table entries give coefficient of the interaction term between education and the Routine Task Intensity index of 2003 occupation. 95% CIs are reported in brackets; SEs are clustered within occupations. See also table 2 for observation counts and further detail on model specifications.

RTI, Routine Task Intensity.

Repeating the analyses using the Frey-Osborne index yields highly similar results across all outcomes and models for men (see and online supplemental tables S1 and S2 in the supplementary materials). For women, the confounder control and sibling models find insubstantial coefficients with 95% CIs covering 0.

## Discussion

### Findings

Using a population data sample that covers a 17-year period, we find that individuals initially employed in occupations with a higher risk of being displaced due to technological change or outsourcing as proxied by RTI are less likely to be employed, and more likely to receive a disability pension or be deceased 16 years later. This also holds in analyses adjusting for individual-level covariates and time-invariant sibling-shared influences, though estimates are substantially attenuated. Allowing the RTI coefficient to differ by educational attainment consistently indicates larger coefficients for those with less education—though some of these interaction terms are imprecisely estimated.

A core strength of our study is the use of administrative data covering the full Norwegian population of salaried employees in November 2003, which gives us a population-representative, attrition-free longitudinal data set of health and employment outcomes across a 16-year follow-up period. In addition, the family linkages and additional population data registers enable analyses controlling for individual educational attainment and family status. The magnitude of the data sample is what allows us to use fixed effect models to control for time-invariant family fixed effects in gender-specific analyses, as such estimates are based exclusively on the outcome differences observed for same-sex siblings employed in occupations with different RTI scores.

The strong and consistent associations seen in our most cautious models, using only within-family variation and controlling for observed sibling differences, is in contrast to results from smaller surveys (such as the German socioeconomic panel) which suggest that those who become unemployed differ in ways that explain later poor health outcomes.^29^ The smaller effect estimates when controlling for confounders and comparing siblings, highlight selection into jobs with a higher risk of automation, consistent with earlier survey-based analyses from Norway.^37^

By conditioning the analysis on the 2003 occupation and using occupational level risk scores, we employ an intention-to-treat design that reduces potential selection effects that would otherwise confound estimates. An analysis comparing those remaining in their occupation to those retraining and finding work in new occupations, for instance, might be comparing individuals whose unobserved differences at baseline explain both their different labour market responses and any difference in outcomes.^38^ The flip side of this benefit, however, is that our study does not allow for a more detailed assessment of potential causal mechanisms involved.

The finding that the influence of RTI tends to be smaller in absolute value for those with higher educational attainment is a good example of this point. Our exposure measure is occupational RTI score in the baseline year, but this reflects the task content of occupations at a specific point in time. Over time, the task content of occupations may shift as new technologies are integrated into existing workflows, and the ease with which this may be achieved may vary with the educational level of the workers. Alternatively, higher educational attainment may signal cognitive skills or personality traits that are broadly valued in the labour market, making it easier for these workers to shift into new occupations. Finally, we might see such gradients if workers with lower educational attainment were more strongly sorted into occupations on the basis of unobserved characteristics. This ‘differential confounding’ explanation, however, would also predict that the gradient should be substantively different in the sibling model that corrects for unobserved confounders at the family level. This does not seem to be the case (figure 2).

The use of occupational risk score measured at baseline in 2003 also needs to be kept in mind when interpreting the results. Many workers will shift occupation over time, and occupations themselves are broad categories covering jobs that may vary in RTI and that may be adjusted over time in response to technological and market forces.^39^ This means that the ‘dose’ of structural risk that different individuals are exposed to over time may differ in ways that our RTI score fails to reflect. There could be substantial opportunities for adaptability of jobs in a period of automation which may reduce unemployment following technological change. Changes in work tasks within occupations may imply that many maintain their job yet change task content. Some high-RTI occupations may also be ‘early stage’ jobs that workers typically progress from over time, in which case the ‘exposure’ measured at baseline will fail to reflect the risk experienced across the observational period. To the extent that this can be viewed as a classical measurement error in our exposure measure, we would expect this to bias our estimates towards 0.

While we would expect qualitatively similar relationships to be present in data from other countries, the magnitude of the effects may be smaller in Norway. Norway is characterised by relatively low economic and social inequality, income levels are high and social security measures are strong. In addition, there are indications that structural risk reshapes labour markets more strongly during economic downturns—and Norway was left relatively unaffected by the 2007 financial crisis and experienced low unemployment throughout our sample period.

## Conclusions

Individuals employed in occupations with high scores on the RTI index, a widely used indicator of how exposed an occupation is to being outsourced or automated, were less likely to remain employed and more likely to receive disability pension or have died 16 years later. The associations were attenuated when educational attainment and family status were controlled for in a family fixed effect model comparing siblings of the same sex. We repeated the analyses using the Frey-Osborne index, which was developed in 2012 to identify occupations at raised risk of being automated as machine-learning techniques improved. This produced similar results for men while the associations for women were small in models with individual-level controls or family fixed effects.

Our findings are consistent with the concern that ongoing automation and outsourcing trends may have negative public health implications for those in affected occupations. If the associations reflect causal effects, these could relate to pathways identified in earlier research, such as increased stress due to employment uncertainty, and to negative consequences of (particularly long term) employment loss on health behaviours. To the extent that they are non-causal, they reveal that the burdens of being employed in occupations in long-term decline—such as increased unemployment and career risks—fall disproportionately on employers with poorer health. Efforts should be made to identify evidence-based policies that can dampen these consequences, for instance, by improving employment adaptability through retraining or career advice programmes.

## Data Availability

Data may be obtained from a third party and are not publicly available.

## References

[1] Voss M , Nylén L , Floderus B , et al . Unemployment and early cause-specific mortality: a study based on the Swedish twin registry. Am J Public Health 2004;94:2155–61. 10.2105/AJPH.94.12.2155 15569968 PMC1448606

[2] Wahrendorf M , Hoven H , Deindl C , et al . Adverse employment histories, later health functioning and national labor market policies: European findings based on life-history data from share and ELSA. J Gerontol B Psychol Sci Soc Sci 2021;76:S27–40. 10.1093/geronb/gbaa049 32322883 PMC8495751

[3] Canivet C , Aronsson G , Bernhard-Oettel C , et al . The negative effects on mental health of being in a non-desired occupation in an increasingly precarious labour market. SSM Popul Health 2017;3:516–24. 10.1016/j.ssmph.2017.05.009 29349242 PMC5769038

[4] Kim W , Park E-C , Lee T-H , et al . Effect of working hours and precarious employment on depressive symptoms in South Korean employees: a longitudinal study. Occup Environ Med 2016;73:816–22. 10.1136/oemed-2016-103553 27540105

[5] Junna L , Moustgaard H , Huttunen K , et al . The association between unemployment and mortality: a cohort study of workplace downsizing and closure. Am J Epidemiol 2020;189:698–707. 10.1093/aje/kwaa010 31976516

[6] Autor DH , Dorn D , Hanson GH . Untangling trade and technology: evidence from local labour markets. Econ J 2015;125:621–46. 10.1111/ecoj.12245

[7] Knapp EA , Bilal U , Dean LT , et al . Economic insecurity and deaths of despair in US counties. Am J Epidemiol 2019;188:2131–9. 10.1093/aje/kwz103 31172197 PMC7212405

[8] Geronimus AT , Bound J , Waidmann TA , et al . Weathering, drugs, and Whack-a-Mole: fundamental and proximate causes of widening educational inequity in U.S. life expectancy by sex and race, 1990-2015. J Health Soc Behav 2019;60:222–39. 10.1177/0022146519849932 31190569 PMC6684959

[9] Bowles J . The computerisation of European jobs. Brussels, Bruegel, 2014.

[10] Wajcman J . Automation: is it really different this time? Br J Sociol 2017;68:119–27. 10.1111/1468-4446.12239 28321856

[11] World Bank . World development report 2016: digital dividends. Washington, DC: The World Bank, 2016.

[12] Brussevich M , Dabla-Norris ME , Khalid S . Is technology widening the gender gap? Automation and the future of female employment: international monetary fund 2019.

[13] Eliason M , Storrie D . Job loss is bad for your health - Swedish evidence on cause-specific hospitalization following involuntary job loss. Soc Sci Med 2009;68:1396–406. 10.1016/j.socscimed.2009.01.021 19243870

[14] Øverland S . Unemployment and mental health. Occup Environ Med 2016;73:717–18. 10.1136/oemed-2016-103831 27492612

[15] Riumallo-Herl C , Basu S , Stuckler D , et al . Job loss, wealth and depression during the great recession in the USA and Europe. Int J Epidemiol 2014;43:1508–17. 10.1093/ije/dyu048 24942142 PMC4190512

[16] Brougham D , Haar J , technology S . Smart Technology, Artificial Intelligence, Robotics, and Algorithms (STARA): Employees’ perceptions of our future workplace. Journal of Management & Organization 2018;24:239–57. 10.1017/jmo.2016.55

[17] Gallie D , Felstead A , Green F , et al . The hidden face of job insecurity. Work, Employment and Society 2017;31:36–53. 10.1177/0950017015624399

[18] McClure PK . “You’re fired,” says the robot: The rise of automation in the workplace, technophobes, and fears of unemployment. Social Science Computer Review 2018;36:139–56.

[19] Nam T . Technology usage, expected job sustainability, and perceived job insecurity. Technol Forecast Soc Change 2019;138:155–65. 10.1016/j.techfore.2018.08.017

[20] Barnay T . Health, work and working conditions: a review of the European economic literature. Eur J Health Econ 2016;17:693–709. 10.1007/s10198-015-0715-8 26280132

[21] Westman M , Etzion D , Horovitz S . The toll of unemployment does not stop with the unemployed. Human Relations 2004;57:823–44. 10.1177/0018726704045767

[22] Lee JO , Jones TM , Yoon Y , et al . Young adult unemployment and later depression and anxiety: does childhood neighborhood matter? J Youth Adolesc 2019;48:30–42. 10.1007/s10964-018-0957-8 30478821 PMC6360094

[23] Montgomery SM , Cook DG , Bartley MJ , et al . Unemployment pre-dates symptoms of depression and anxiety resulting in medical consultation in young men. Int J Epidemiol 1999;28:95–100. 10.1093/ije/28.1.95 10195671

[24] Kaspersen SL , Pape K , Ose SO , et al . Unemployment and initiation of psychotropic medication: a case-crossover study of 2 348 552 Norwegian employees. Occup Environ Med 2016;73:719–26. 10.1136/oemed-2016-103578 27165811

[25] Min J-young , Lee K-jong , Park J-beom , et al . Social engagement, health, and changes in occupational status: analysis of the Korean longitudinal study of ageing (KLoSA). PLoS One 2012;7:e46500. 10.1371/journal.pone.0046500 23056323 PMC3462751

[26] Melchior M , Berkman LF , Niedhammer I , et al . Social relations and self-reported health: a prospective analysis of the French Gazel cohort. Soc Sci Med 2003;56:1817–30. 10.1016/S0277-9536(02)00181-8 12639598

[27] Burdorf A . The importance of solid employment for health. Scand J Work Environ Health 2008;34:81–2. 10.5271/sjweh.1216 18470436

[28] Waddell G , Burton AK . Is work good for your health and well-being? The Stationery Office, 2006.

[29] Kaiser M , Bauer JM , Sousa-Poza A . Does unemployment lead to a less healthy lifestyle? Appl Econ Lett 2017;24:815–9. 10.1080/13504851.2016.1231888

[30] Goos M , Manning A , Salomons A . Explaining job polarization: Routine-biased technological change and offshoring. Am Econ Rev 2014;104:2509–26. 10.1257/aer.104.8.2509

[31] Frey CB , Osborne MA . The future of employment: how susceptible are jobs to computerisation? Technol Forecast Soc Change 2017;114:254–80. 10.1016/j.techfore.2016.08.019

[32] Acemoglu D , Autor D . Skills, tasks and technologies: Implications for employment and earnings. In: Handbook of labor economics. Elsevier, 2011: 1043–171.

[33] Mihaylov E , Tijdens KG . Measuring the routine and Non-Routine task content of 427 Four-Digit ISCO-08 occupations, 2019. 10.2139/ssrn.3389681

[34] Nordic Burden of Disease Collaborators . Life expectancy and disease burden in the Nordic countries: results from the global burden of diseases, injuries, and risk factors study 2017. Lancet Public Health 2019;4:e658–69. 10.1016/S2468-2667(19)30224-5 31759894 PMC7098475

[35] Hemmings P , Prinz C . Sickness and disability systems: comparing outcomes and policies in Norway with those in Sweden the Netherlands and Switzerland 2020.

[36] Bratsberg B , Raaum O , Sørlie K . Foreign-Born migration to and from Norway. International migration, economic development and policy 2007:259–91.

[37] Hessel P , Christiansen S , Skirbekk V . Poor health as a potential risk factor for job loss due to automation: the case of Norway. Occup Environ Med 2018;75:227–30. 10.1136/oemed-2017-104349 29030397

[38] Gupta SK . Intention-To-Treat concept: a review. Perspect Clin Res 2011;2:109. 10.4103/2229-3485.83221 21897887 PMC3159210

[39] Arntz M , Gregory T , Zierahn U . Revisiting the risk of automation. Econ Lett 2017;159:157–60. 10.1016/j.econlet.2017.07.001

