# Supplementary material for: Technology-induced job loss risk, disability and all-cause mortality in Norway
Source: Occup Environ Med. 2021 Sep 24;79(1):32–7. doi: 10.1136/oemed-2021-107598 (PMC8685638; doi:10.1136/oemed-2021-107598)
Supplement: Supplementary data [file oemed-2021-107598supp001.pdf]

Supplementary materials

Frey and Osborne

Results of performing the same analyses as those shown in the main manuscript, using the Frey-Osborne index in place of the Routine Task Intensity index as an indicator of technology-induced risk of job loss.

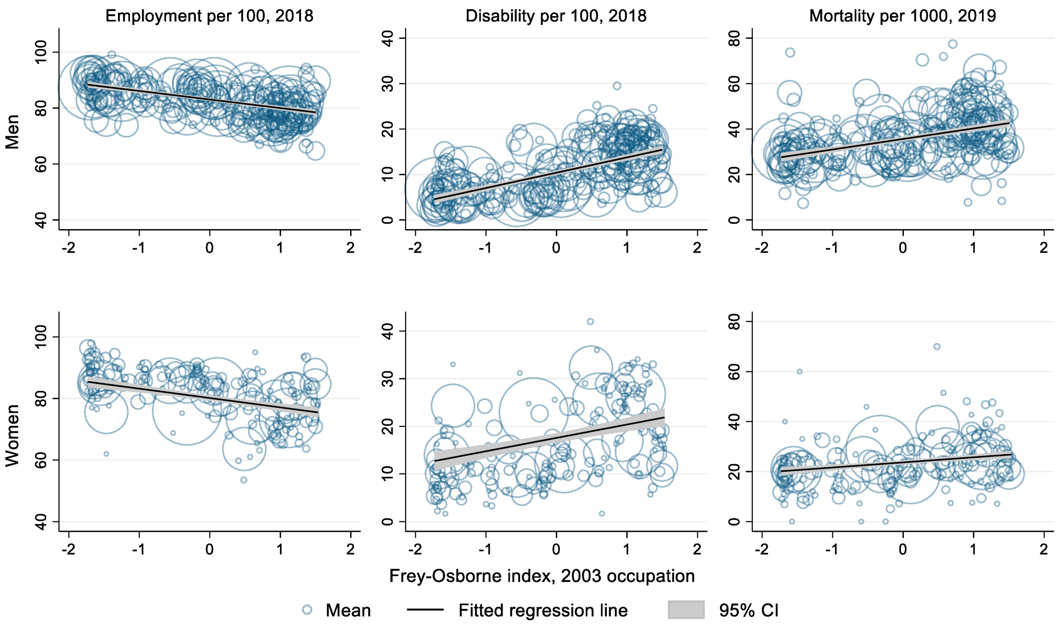

Figure S-1 – Occupational risk score 2003 and employment, disability, and mortality 2018 and 2019. Scatter points show the average outcome in 2018 and 2019 versus the Frey-Osborne Index for each of 246 (men) and 185 (women) 2003 occupations. Occupations with higher Frey-Osborne scores are expected to be more influenced by automation and globalization. Scatter points are weighted by the observation count of the 2003 occupation; cells with fewer than 100 observations are omitted from the figure. The Frey-Osborne Index is standardized to have mean zero and standard deviation one in the 2003 workforce. Slope [95% CI] of regression lines are -3.1 [-3.6, -2.5], 3.4 [2.9, 3.8], and 4.6 [3.7, 5.6] in the top panels, and -3.0 [-3.9, -2.2], 2.8 [1.8, 3.8], and 2.0 [1.2, 2.9] in the bottom panels.

Table S-1 – Regression results, coefficient of Frey-Osborne index of 2003 occupation.

	Men			Women		
	Model w/o controls (1)	Model with controls (2)	Sibling model (3)	Model w/o controls (4)	Model with controls (5)	Sibling model (6)
Dependent variable:						
Employment per 100, 2018	-3.1<br>[-3.7, -2.4]	-1.6<br>[-2.1, -1.1]	-1.4<br>[-1.8, -1.1]	-3.1<br>[-4.8, -1.5]	-0.7<br>[-1.6, 0.2]	-0.5<br>[-1.0, 0.1]
Disability per 100, 2018	3.4<br>[2.8, 3.9]	1.7<br>[1.4, 2.1]	1.4<br>[1.1 ,1.7]	2.9<br>[0.8, 4.9]	0.4<br>[-1.0, 1.6]	0.2<br>[-0.3, 1.1]
Mortality per 1000, 2019	4.6<br>[3.5, 5.7]	2.1<br>[1.3, 2.9]	2.2<br>[1.1, 3.3]	2.1<br>[1.2, 3.1]	0.6<br>[-0.5, 1.7]	0.4<br>[-1.0, 1.9]
Observations	416 003	416 003	186 369	376 413	376 413	158 524
Number of families			83 715			71 647
Control variables	None	Age, education, civil status, childless	Age, education, civil status, childless, family fixed effects	None	Age, education, civil status, childless	Age, education, civil status, childless, family fixed effects
Sample	All	All	Brothers	All	All	Sisters

Note: Table entries give change in dependent variable from a one standard deviation increase in the Frey-Osborne index of 2003 occupation. 95% confidence intervals are reported in brackets; standard errors are clustered within occupations. Models in columns (2), (3), (5) and (6) include indicator variables for 20 ages and 13 levels of educational attainment, as well as indicator variables for single status and childlessness in 2003.

Table S-2 – Coefficient of interaction term between Frey-Osborne index of 2003 occupation and educational attainment.

Dependent variable:	Employment per 100, 2018		Disability per 100, 2018		Mortality per 1000, 2019	
Sample:	All<br>(1)	Siblings<br>(2)	All<br>(3)	Siblings<br>(4)	All<br>(5)	Siblings<br>(6)
A. Men	0.3<br>[0.1,0.4]	0.2<br>[0.1,0.4]	-0.5<br>[-0.6,-0.4]	-0.4<br>[-0.5,-0.3]	-0.8<br>[-1.0,-0.5]	-0.7<br>[-1.2,-0.3]
B. Women	0.3<br>[0.0,0.6]	0.2<br>[-0.0,0.3]	-0.4<br>[-0.8,0.0]	-0.2<br>[-0.4,0.0]	-0.4<br>[-0.7,-0.2]	-0.2<br>[-0.7,0.2]
Control variables	Frey-Osborne index, education, age, civil status, childless	Frey-Osborne index, education, age, civil status, childless, family fixed effects	Frey-Osborne index, education, age, civil status, childless	Frey-Osborne index, education, age, civil status, childless, family fixed effects	Frey-Osborne index, education, age, civil status, childless	Frey-Osborne index, education, age, civil status, childless, family fixed effects

Note: Table entries give coefficient of the interaction term between education and the Frey-Osborne index of 2003 occupation. 95% confidence intervals are reported in brackets; standard errors are clustered within occupations. See also Table A-1 for observation counts and further detail on model specifications.

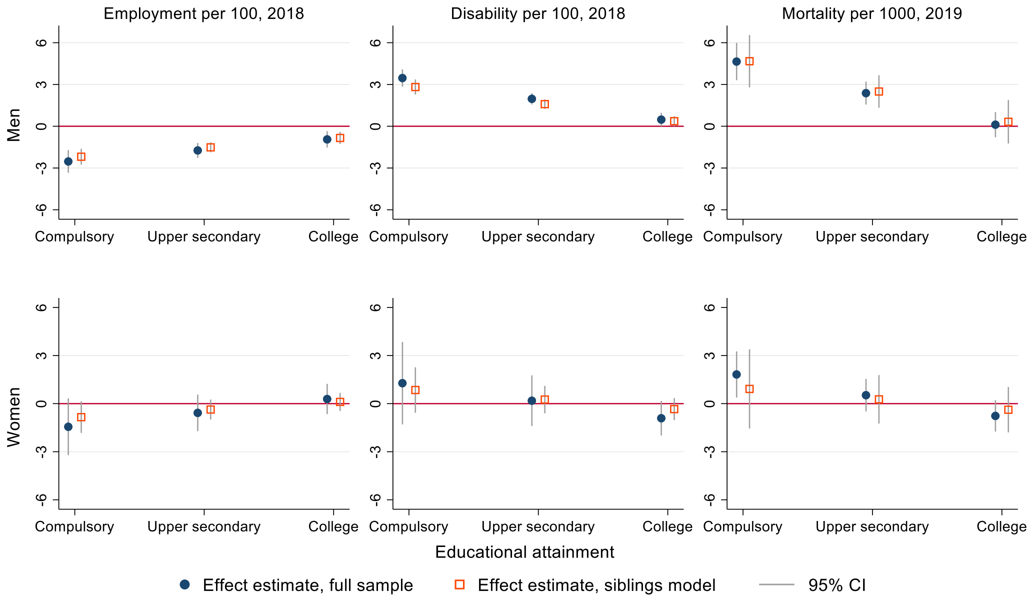

Figure S-2 – Effect estimates of Frey-Osborne index on employment and health outcomes by educational attainment. Scatter points show the estimated effects of a one standard deviation increase in the Frey-Osborne index of 2003 occupation, evaluated at educational attainments of compulsory schooling, completed upper secondary, and college. Estimates are based on regression models where attainment is interacted with the Frey-Osborne index. Regression models control for age (20 levels), single status, and childlessness in 2003. Sibling models add family fixed effects. Standard errors are clustered within 2003 occupation.

Variables and estimation code

The following variables are used in the study:

- Outcome variables:
    - Employment: Indicator variable for whether the sum of earnings from registered employment exceeds 1G (the base unit of the Norwegian pension system, and the minimum earnings level required to earn pension rights) – using data from the registers of the tax and welfare administrations. In 2018, 1G equaled 95 800 NOK (approximately 10 000 Euros)
    - Disability: Registered as recipient of disability pension at any level (i.e., including those with reduced but some residual work capacity)
    - Mortality: Marked as deceased in population registry
- Explanatory variables:
    - Sex
    - Year of birth
    - Age: Integer age Dec 31, 2003
    - Educational attainment: 13 levels of years of schooling defined by the six-digit code of the individual's highest completed education in 2003, as recorded in the National Education Data Base ("NUDB"). The administrative data use the Norwegian Standard Classification of Education (NUS2000), as further documented here: https://www.ssb.no/en/klass/klassifikasjoner/36/
    - Civil status: Single is defined by having no registered partner or cohabitant at the end of 2003. Details: https://www.ssb.no/en/omssb/tjenester-og-verktoy/data-til-forskning/befolkning/data-om-familie-samboere-og-ektefeller
    - Childlessness: No registered children in the population registry as of Dec 31, 2003
    - Mother identifier: Mother's encrypted personal identity number, as recorded in the population registry
    - Occupation: 4-digit occupation code (ISCO-88 standard) recorded in the employer-employee register
    - Routine Task Intensity index: Measure computed from occupational task scales downloaded from the O*NET data base, version 5.0, with cross-walks from SOC codes to ISCO-88 codes downloaded from the US Bureau of Labor Statistics (https://www.bls.gov/emp/documentation/crosswalks.htm) and Statistics Norway (https://www.ssb.no/en/klass/klassifikasjoner/145/korrespondanser). We compute the RTI index as the sum of O*NET scores on manual routine and cognitive routine scales, and subtract the sum of scores on the non-routine manual, analytical, and interpersonal task scales.
    - Frey-Osborne index: Data collected from appendix of Frey and Osborne (2017).

Stata code for estimation:

```
#delimit ;

set more 1;


log using reg_tables, t replace;

*fig 1, occupation-by-gender data;

use data\occupation_data, clear;

forvalues s= 0/1 {;
foreach y of var yemp2018 ydis2018 ymort2019 {;
di "sex= " `s' " dep var " `"`y'"';
reg `y' std_rti if sex==`s' & ncell>99 [aw=ncell];
};
};



*table 2, micro data;

use data\micro_data, clear;

forvalues s= 0/1 {;
foreach y of var emp2018 dis2018 mort2019 {;
di "sex= " `s' " dep var " `"`y'"';
di "model 1, no controls";
reg `y' c.std_rti if sex==`s', cl(isco88);
di "model 2, with controls";
reghdfe `y' c.std_rti i.single i.cldlss if sex==`s', absorb(age educ)
cl(isco88);
di "model 3, add family fixed effects";
reghdfe `y' c.std_rti i.single i.cldlss if sibsmpl==1 & sex==`s',
absorb(morid age educ) cl(isco88);
};
};

log close;
```
